# Subcellular localization of Mayven following expression of wild type and mutant EGFP tagged cDNAs

**DOI:** 10.1186/1471-2202-11-63

**Published:** 2010-05-26

**Authors:** Paul Montague, Peter GE Kennedy, Susan C Barnett

**Affiliations:** 1 Division of Clinical Neuroscience, Glasgow Biomedical Research Centre, Room 4B17, 120 University Place, University of Glasgow, Glasgow G12 8TA, UK; 2 Division of Clinical Neurosciences, Southern General Hospital, University of Glasgow, Glasgow G54 5TF, UK

## Abstract

**Background:**

Process formation by glial cells is crucial to their function. Mayven, an actin binding, multi-domain polypeptide, and member of the BTB-BACK-Kelch family have been shown to be important in oligodendrocyte process extension. To assess the role of Mayven in neural cell process extension we have tracked the subcellular distribution of exogenous Mayven following expression of a rat Mayven -EGFP cDNA in a variety of neural cell backgrounds and specifically in OEC tranfectants following drug treatment to disrupt the integrity of the cytoskeleton. A comparison was made between the subcellular localization following transient transfection of OECs with full-length Mayven cDNA and a series of mutant domain constructs.

**Results:**

The subcellular location of Mayven in OEC transfectants showed a characteristic distribution with intense foci of staining towards the process tips corresponding to regions of accumulated Mayven overlapping in part with lammelipodial actin and was absent from the filipodia and the outer membrane. This signature pattern was also observed in Schwann cells, Oli-Neu cells, astrocytes and the neuroblastoma cell line B104 transfectants and resembled the exogenous and endogenous Mayven distribution in oligodendrocytes. This contrasted with the localization pattern in non-neural cells. There was a re-localization of Mayven in OEC transfectants following drug treatment to challenge the integrity of the actin cytoskeleton while breakdown of the microtubular component had no discernible impact on the accumulation of Mayven in the process tips. Deletion of the first three amino acids of the SH3 motif of the putative Fyn Kinase binding domain at the amino terminus significantly compromised this signature pattern as did the removal of the last Kelch repeat unit of six unit Kelch domain comprising the carboxyl terminus. In addition, there was a reduction in process length in mutant transfectants. Co-expression studies with a haemagglutinin (HA) tagged wild type Mayven cDNA and EGFP tagged mutant cDNAs suggested a homomeric interaction mediated by the BTB/POZ domain.

**Conclusions:**

Exogenous Mayven is transported to the lamellipodia in neural transfectants associating with the actin cytoskeletal network. In addition to the importance of the internal BTB/POZ domain, this subcellular distribution pattern is dependent on the presence of an intact amino and carboxyl terminus.

## Background

Actin polymerization driven membrane protrusion, initially results in the formation of fine filipodia projections comprising parallel actin bundles. The filipodia widen to form lamellipodia by creating a branched mesh network of orthogonal actin. Consequent recruitment of microtubules provides mechanical stability to the extending lamellipodia. This is a common mechanism shared by a range of process bearing cell types [1] including oligodendroglia [2] and olfactory ensheathing cells (OECs) [3,4]. The exact mechanisms by which cells extend processes and carry out complex cell:cell interactions, for example how oligodendrocytes enwrap axons and form myelin sheaths are not known. However, it is apparent that to generate these vast, highly organised membranous extensions, extensive coordinated cytoskeletal remodelling must occur [5]. Consequently, the contribution of cytoskeletal dynamics to process extension and axonal wrapping is fundamental to myelination. Identifying the molecules involved and understanding their mechanism of action is crucial to extend our knowledge of how myelin formation occurs during development and remyelination.

**Figure 1:**
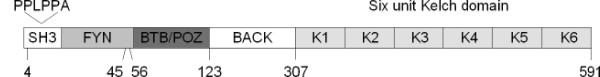
**Domain organization of Mayven**. Amino acid positions demarcating the individual domains are numbered and the sequence of the SH3 motif is highlighted.

Recently, a candidate protein, Mayven (KLHL2) has been reported to have a crucial role in the extension of oligodendrocyte precursor cell (OPC) processes [6,7]. This actin binding protein [8] belongs to the BTB-BACK-Kelch family with around 50 members [9]. Although these proteins are highly conserved at the structural domain level, they have a wide range of cellular functions including the stability and dynamics of microfilaments [10,11]. The predicted 593 residue Mayven polypeptide has four structural domains (Fig. 1). The presence of a Fyn Tyrosine Kinase Binding domain (AAs 4-45) including the proline rich SH3 ligand motif (AAs 4-10) (P_1_P_2_LP_3_P_4_A) is intriguing considering the crucial role ascribed to Fyn Kinase in myelinogenesis and in differentiation of cultured oligodendrocytes [12,13]. Co-immunoprecipitation and GST pull-down experiments confirm an association between the SH3 motif of Mayven and Fyn Kinase [7]. The **B**road complex, **T**ramtrac, **B**ric-a-brac)/(**Po**xvirus, **Z**inc finger (BTB/POZ) domain (AAs 56-123) can self associate or heteromerize with other BTB/POZ proteins [14,15]. AAs 124-307 encode a **B**TB **a**nd **C**-terminal **K**elch (BACK) domain with as yet no confirmed function [16]. The Kelch domain at the carboxyl terminus (AAs 308-591) is associated F-actin binding [8] and comprises six contiguous units forming a characteristic β-propeller tertiary structure exposing a plethora of contact sites for interacting side chains of as yet unidentified binding partners [17]. This domain organization may favour the formation of a multi-protein complex integral to cytoskeletal remodelling. A precedent for this has been reported by laboratory colleagues showing that Krp1, a BTB-BACK-Kelch family member forms a multi-protein complex during tumour invasion leading to pseudopodial elongation. Krp1 binds to the actin binding protein Lasp-1 at the leading edge of the membrane interacting with an integrin CD44-adaptor protein Ezrin complex providing a molecular conduit between the ECM and intracellular signal transduction [18].

We and others have shown, using immunocytochemistry, that Mayven is present in oligodendrocytes and OPC processes; especially in the retracting end of the cell, the leading edge of lamellipodia and at intervals along the length of some processes [6,7]. This pattern correlates with areas where dynamic reorganisation of actin occurs and where process formation is ongoing. Using RNA interference (RNAi) we demonstrated that Mayven has a role in process extension in OPCs but does not affect their migration or differentiation [6]. Confirmation of our siRNA effect was demonstrated by others, who also showed that over expression of a Mayven cDNA construct in an OPC line led to an increase in both process outgrowth and average process length, and conversely, OPC process extension was inhibited following microinjection of Mayven antibodies [7]. Earlier, a study by Soltysik-Espanola et al [8], reported co-localization of endogenous Mayven and cortical actin in U373-MG astrocytoma/glioblastoma cells in the cell body and along the processes of rat hippocampal neurons.

In addition to Mayven, other proteins are known to regulate OPC processes extension. Following engagement by ECM molecules such as fibronectin and laminin, the differentially expressed αvβ1 and αvβ5 integrins can trigger several signalling cascades by activation of Fyn Kinase and the small GTPase family members, RhoA, Rac1 and Cdc42 [19,20] linking extracellular stimuli to changes in the organization of the cytoskeleton. Activation of the actin nucleation complex Arp2/3 is the rate limiting step in actin polymerization driven membrane protrusion [1]. This is mediated in part by binding N-WASP following activation by Cdc42 while Rac1 activated WAVE1 binds to Arp2/3 leading to filipodia and lamellipodia formation respectively [21]. Like Mayven, these polypeptides have been shown by immunostaining to localize to the OPC lamellipodium and the leading edge of Schwann cells [2]. Recently another BTB-BACK-Kelch protein originally termed KLHL1 [22] that is specifically found in the brain, is transported to the leading edge in the OPC, where it co-localizes with actin [23]. Antisense and overexpression studies suggest a key role for this protein in process extension. Given the similarity to Mayven, the authors refer to this protein as **M**ayven **R**elated **P**rotein 2 (MRP2).

The aims of this study were two fold. First, to explore the relationship between Mayven and the cytoskeleton by examining the subcellular localization of EGFP tagged Mayven following drug treatment to challenge the integrity of the cytoskeletal network. Second, if Mayven does have a critical role in actin polymerization driven neural membrane protrusion, the protein is likely to be transported to its correct site for interaction with as yet unidentified components of the process extension machinery. The Fyn Kinase binding domain with its close association with glial development and myelinogenesis [12,13] and the actin binding properties of the Kelch domain [8] may have functional roles in the regulated transport and accumulation of Mayven in the process tips. To identify cognate sequences involved in this process, we have tracked the subcellular destination of exogenous tagged Mayven following transient transfection of OECs with a range of amino and carboxyl Mayven domain mutants. Although this cell type has a highly dynamic morphology, two distinct morphologies predominate. Those with a spindle like appearance with long processes which can extend over considerable distances and a population with a more flat cell body and shorter thick processes [24]. Time-lapse imaging studies have reported that individual OECs continually change their shape, size and direction of movement [3,4]. In addition, compared to oligodendroyte lineage cells, OECs have a high transfection rate. This well established experimental paradigm can provide insight into the function of the endogenous counterpart as highlighted in a recent study which compared the distribution of EGFP tagged wild type *Myelin Protein Zero *cDNA to that of several mutant forms following transient transfection [25].

## Methods

### Constructs

**Figure 2:**
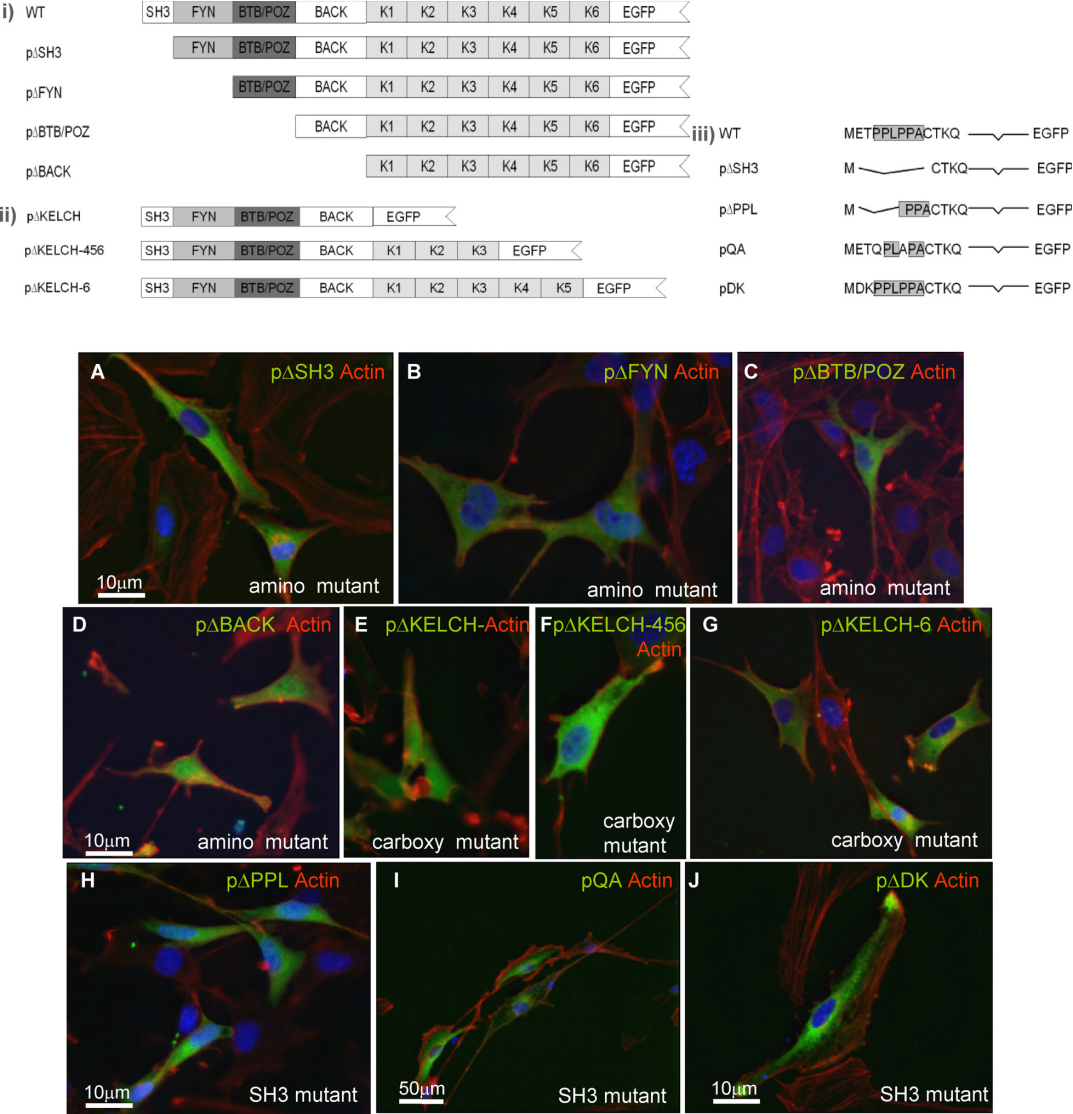
**Subcellular distribution pattern of exogenous Mayven following expression of a panel of pMayven-EGFP mutants**. **Group (I) Amino mutants, Group (II) Carboxyl mutants and Group (III) SH3 motif mutants**. OECs were transfected with three series of mutant cDNAs: amino terminus deletion constructs **(A-D)**, carboxyl terminus deletion constructs **(E-G)**, SH3 motif mutants **(H-J) **and actin stained. Only pDK transfectants **(J) **gave a wild type distribution pattern.

The 1782 bp rat Mayven cDNA corresponding to the 593 amino acid Open Reading Frame [6] cloned in pPCR-ScriptCamSK (Stratagene) was used as a template for the construction of wild type Mayven, a panel of amino and carboxyl domain deletion mutants and a set of mutants with a disrupted SH3 motif of the Fyn Kinase binding domain (Fig. 2(i),(ii),(iii)). Using restriction site anchored PCR primers pairs (Table 1), amplicons were synthesized using the proofreading EasyA Polymerase (Stratagene) which possesses both terminal transferase and exonuclease activities to generate high-fidelity PCR products containing 3'-A overhangs and cloned into the T/A vector pSC-A (Stratagene). The inserts were released by restriction enzyme digestion and covalently linked in-frame at the corresponding restriction sites in the Multiple Cloning Site (MCS) of the commercial carboxyl reporter vector pEGFP-N1 encoding the red-shifted variant of the green fluorescent protein (EGFP) (BD Biosciences). In addition, wild type Mayven cDNA was subcloned into the amino reporter vector pEGFP-C3 (Invitrogen) and pMH (Roche) encoding a nine amino acid haemagglutinin (HA) epitope at the carboxyl terminus. Forward primers for cloning into pEGFP-N1 and pMH in which the respective reporters are in the carboxyl orientation, contained an artificial Kozak sequence to increase fusion protein levels. As depicted in Table 1, common primers (see dagger symbol) were used in the construction of the series of Mayven cDNA mutants. All recombinants were verified by sequencing to ensure in-frame integrity at the Mayven-EGFP junction.

**Table 1:** Sequence of PCR cloning primers

Construct	Forward primer^1^	Reverse Primer ^1^
pMayven-EGFP	(*Bgl*II)5'-**AGATCT**CCCACCATGGAGACGCCGCCGCTGCCT-3'	(*Hind*III) 5'-**AAGCTT**TAATGGTTTATCAATAACTGT-3'

pΔSH3	(*Bgl*II) 5'-**AGATCT**CCCACCATGTGCACAAAGCAGGGTCATCAG-3'	(*Hind*III) 5'-**AAGCTT**TAATGGTTTATCAATAACTGT-3' **†**

pΔFYN	(*Bgl*II) 5'-**AGATCT**CCCACCATGAACGAACTAAGAAGTCAA-3'	(*Hind*III) 5'-**AAGCTT**TAATGGTTTATCAATAACTGT-3' **†**

pΔBTB/POZ	(*Bgl*II) 5'-**AGATCT**CCCACCATGGTCCTCCTCCCAGCAGCTGGC-3'	(*Hind*III) 5'-**AAGCTT**TAATGGTTTATCAATAACTGT-3' **†**

pΔBACK	(*Bgl*II) 5'-**AGATCT**CCCACCATGAACCTTCCCAAATTGATG-3'	(*Hind*III) 5'-**AAGCTT**TAATGGTTTATCAATAACTGT-3' **†**

pΔKELCH	(*Bgl*II)5'-**AGATCT**CCCACCATGGAGACGCCGCCGCTGCCT-3' **†**	(*Hind*III) 5'-**AAGCCT**TTTGGGAAGGTTCATGGGTGT-3'

pΔKELCH-456	(*Bgl*II)5'-**AGATCT**CCCACCATGGAGACGCCGCCGCTGCCT-3' **†**	(*Hind*III) 5'-**AAGCCT**ACCTCCAACAACGCCAACGCC-3'

pΔKELCH-6	(*Bgl*II)5'-**AGATCT**CCCACCATGGAGACGCCGCCGCTGCCT-3' **†**	(*Hind*III) 5'-**AAGCCT**ACCATTAACTGCGCAGACACC-3'

pΔPPL	(*Hind*III) 5'-**AGATCT**CCCACCATGCCTCCCGCATGCACAAAGCAG-3'	(*Hind*III) 5'-**AAGCTT**TAATGGTTTATCAATAACTGT-3' **†**

pQA	(*Hind*III) 5'-**AGATCT**CCCACC ATGGAGACGCAGCCGCTGCCTGCCGCA-3'	(*Hind*III) 5'-**AAGCTT**TAATGGTTTATCAATAACTGT-3' **†**

pDK	(*Hind*III)5'-**AGATCT**CCCACCATGGACAAGCCGCCGCTGCCTCCCGCA-3'	(*Hind*III) 5'-**AAGCTT**TAATGGTTTATCAATAACTGT-3' **†**

pEGFP-Mayven	(*Xho*I) 5'-**CTCGAG**ATGGAGACGCCGCCGCTGCCT-3'.	(*Hind*III) 5'-**AAGCTT**TAATGGTTTATCAATAACTGT-3'

pEGFP-ΔFYN	(*Xho*I) 5'-**CTCGAG**-ATGAACGAACTAAGAAGTCAA-3'.	(*Hind*III) 5'-**AAGCTT**TAATGGTTTATCAATAACTGT-3'

pMayven-HA	(*Hind*III) 5'-**AGCTT**CCCACCATGGAGACGCCGCCGCTGCCT-3'	(*Kpn *I) 5'-**GGTACC**TAATGGTTTATCAATAACTGT-3'.

^1 ^Restriction enzyme sites are italicised and corresponding sequences are in bold. The artificial Kozak motif is underlined.

**† **Common primers used in the construction of the three sets of Mayven mutants.

pLasp-1-GFP and pLasp-1-Cherry were kindly supplied by Dr Heather Spence (Beatson Institute). pEGFP-Actin (BD Biosciences) encodes a fusion protein comprising the green fluorescent protein cDNA and the human cytoplasmic β-actin cDNA.

### Reverse Transcription Polymerase Chain Reaction (RT.PCR)

Cells grown on 13 mm PLL coated coverslips or PLL treated T25 cm^2 ^flasks were rinsed once in PBS and homogenized in 1 ml of RNABee (ams Biotechnology) and total cellular RNA extracted according to manufacturer's guidelines. Random hexamer (Invitrogen) primed cDNA synthesis was performed on 2 μg of total cellular RNA prepared from T25 cm^2 ^flasks or 250 ng isolated from 10^5 ^cells seeded onto coverslips using SuperScript III ™ (Invitrogen) reverse transcriptase following supplier's guidelines. PCRs were carried out on 5 ng of cDNA using REDTaq ReadyMix PCR Reaction Mix (Sigma). Routine thermal cycling conditions were as follows: an initial denaturation step of 94°C/5 mins, a core cycle comprising (94°C/1 min-55°C/1 min-72°C/1 min) for 25-35 cycles followed by a final extension of 72°C/10 mins. Rat primer sequences and product lengths were as detailed below. *β-actin *: Forward (5'-CATTGCTGACAGGATGCAGAAGGA-3'), Reverse (5'-ACTCATCGTACTCCTGCTTGCTGA-3') product length 146 bps. *Cyclophilin *: Forward (5'-ACCCCACCGTGTTCTTCGAC-3'), Reverse (5'-CATTTGCCATGGACAAGATG-3') product length 300 bps. *Mayven *: Forward (5'-ACCCTGTCAACTGCTTAGG-3'), Reverse (5'-GCTGAAAATGTCCTTCAGGC-3') product length 1323 bps. *Cyclophilin *and *β-actin *primers were included as internal controls for invariant gene expression. PCR products were visualised on ethidium bromide stained 2% agarose gels and images captured on an UVIdocD55XD system (Uvitec UK).

### Cell culture and transfection

A range of neural cell types were used. An OPC line generated from the optic nerve of 7-day old rat pups [26] was immortalised with the c-myc gene (O2A-cmyc) and maintained as OPCs in 40% B104 conditioned medium/SATO (neuroblastoma cells that secrete growth factors that promote OPC proliferation [27] or PDGF and FGF2 20 ng/ml of each (Peprotech UK). These cells (referred to as OPCs) differentiate after the removal of growth factors with the subsequent elaboration of many processes typical of mature oligodendrocytes [26,28]. The second OPC line used, referred to as rat Oli-Neu were grown in 1-2% horse serum/SATO [29]. We also studied glial cells that had a larger cytoplasm with more visible cytoskeletal proteins, eg rat olfactory ensheathing cells (OECs,) [24] and cortical astrocytes which were isolated and maintained as previously described [30]. B104 cells grown in 10% FBS/DMEM were also used. Two non-neural cell types were used for comparison; Cos7 and BHK fibroblasts which were maintained in 10%FBS/DMEM. Neural cells selected for transient transfections were plated onto poly-L-Lysine (PLL, 13 μg ml^-1 ^Sigma, Poole UK) coated 13 mm glass coverslips at a sub confluent cell density and incubated overnight in their appropriate medium. The following day 0.5 μg plasmid DNA transient transfections were performed using Lipofectomine (Ltx) in conjunction with PLUS reagent following the manufacturer's guidelines (Invitrogen, UK). Exposure times to the Ltx complex varied from four hrs to overnight dependent on the toxicity to the reagent, which was cell type variable. Post-treatment, the cells were washed once in PBS, fixed in pre-warmed 4% paraformaldehyde for ten min and washed three times in pre-warmed PBS.

### Immunostaining

The primary antibodies, mouse HA monoclonal antibody IgG1HA.11 Clone16B12 (Convance) and mouse acetylated tubulin monoclonal antibody IgG2b (Sigma) were used at 1: 5000 and 1: 500 respectively. Fixed and washed coverslips as previously described were treated in cold methanol for 10 min, washed three times in PBS and treated overnight with primary antibody diluted in 1% normal goat serum/PBS. The coverslips were rinsed three times in PBS and treated with FITC and TRITC fluorochrome-conjugated secondary antibodies (Southern Biotechnique) at 1:100 diluted in PBS for 45 min at room temperature. The coverslips were washed three times in PBS, rinsed briefly in distilled water and mounted in Vectashield with DAPI. The cells were visualized by fluorescence microscopy (Zeiss Axioskop) using MetaMorph image analysis from Molecular devices. For confocal analysis of Mayven and actin expression OECs were visualized using a Zeiss LSM 510 Meta equipped with a multi-line Argon laser and two HeNe lasers.

### Cytoskeletal staining

Actin was detected with Texas Red conjugated Phalloidin (Invitrogen). The fixed cells were permeablised with 0.01% TritonX-100/PBS for five min at room temperature. Following three PBS washes the coverslips were incubated with 1% BSA/PBS for 20 min at room temperature. Each coverslip was treated with 2.5 μl of a 6.6 μM methanolic stock of Phalloidin Texas Red in 100 μl of 1% BSA/PBS for 20 min at room temperature, rinsed three times in PBS, twice in water and mounted in Vectashield with DAPI. The microtubular network was stained with a mouse monoclonal antibody raised against acetylated tubulin (IgG2b, Sigma).

### Pharmacolological perturbation of the OEC cytoskeleton

*i) Cytochalasin *B. Cytochalasin B (Sigma) which induces microfilament disassembly was reconstituted as a 1 mM stock in ethanol. OECs were seeded onto coverslips in the presence of 0.5 μM cytochalasin B for 18 hr prior to a four hr transient transfection. Post transfection, the medium was aspirated, washed twice with PBS and incubated for a further 14 hrs in the presence of 0.5 μM Cytochalasin B, fixed and actin stained with Texas Red Phalloidin to assess microfilament disruption.

*ii) Wiskostatin*. Wiskostatin blocks actin filament assembly by inhibiting the formation of the Arp2/3 nucleation complex. The drug was reconstituted as a 10 mM stock. Transiently transfected OECs 24 hrs post transfection were treated with a range of Wiskostatin concentrations up to 10 μM for 2 hrs, fixed and actin stained as described elsewhere.

*iii) Nocodazole*. The microtubule depolymerisation agent Nocodazole (Sigma) was reconstituted in DMSO as a 10 mM stock. Transiently transfected OECs on coverslips 24 hrs post transfection were treated with 30 μM for three hrs Nocodazole. The cells were fixed and immunostained with the acetylated tubulin antibody to assess microtubular breakdown.

### Measurement of OEC process length

Relative process length expressed in terms of arbitrary pixel unit values was measured using the NIH Image J system. Measurements were confined to non-transfected and transfected cells with a spindle like morphology which had two or three well defined processes. The length of each process from the centre of the nucleus to the tip was recorded for each individual cell. The number of process measurements for each grouping was as follows: 23 non-transfected cells, 12 wild type transfectants, 6 pDK transfectants and 75 mutant transfectants from a pool comprising pQH, pΔSH3, pΔFYN, pΔBTB/POZ, pΔBACK, pΔKELCH, pΔKELCH-456 and pΔKELCH-6. The statistical significance was assessed by a One-way Anova Test followed by a Dunnett's Multiple Comparison Test.

### Time-lapse microscopy

Transfectants were visualised over an 18 hr period. Images were captured every 3 min using the Nikon TE2000 time-lapse microscope that has a Nikon perfect focus (PFS) and images acquired using Metamorph 7 software (Molecular Devices). OPCs were plated onto PLL treated glass well dishes (Iwaki). When 50% confluent, the plate was positioned in a controlled temperature and CO_2 _chamber, 20 cells were selected for time lapse. Images were collected every 3 mins for 18 hrs and the data converted to a movie (AVI format) or saved as individual stack files using Metamorph.

## Results

### Subcellular distribution of exogenous Mayven in transfected neural and fibroblast cells

**Figure 3:**
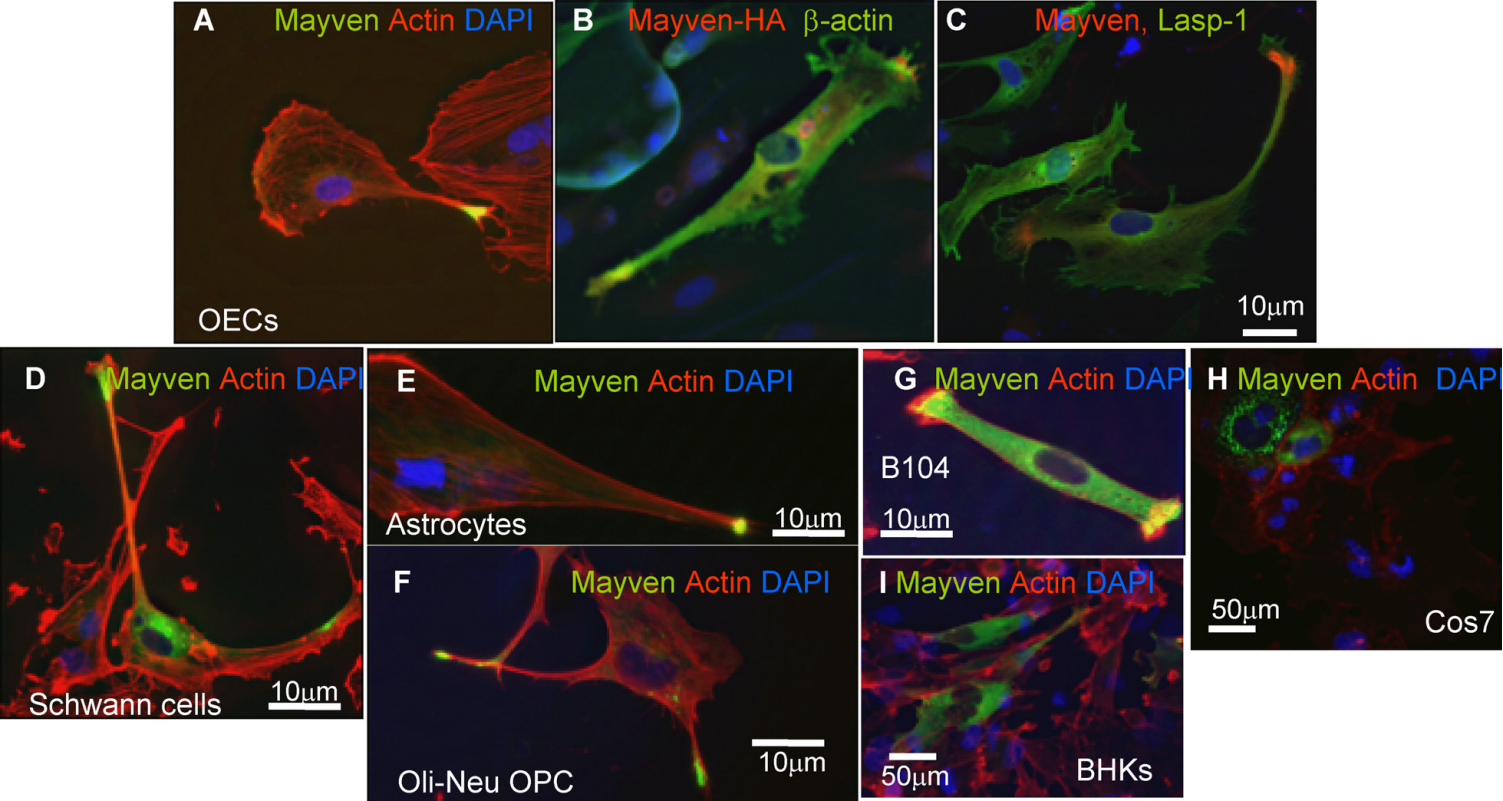
**Subcellular distribution pattern of exogenous Mayven in neural and non-neural cells**. **(A) **pMayven-EGFP/OECs, **(B) **(pMayven-HA/pEGFP-βActin)/OECs, **(C) **(pMayven-HA/pLasp-EGFP)/OECs. **(D) **Schwann cells, **(E) **astrocytes, **(F) **Oli-Neu cells, **(G) **B104 cells, **(H) **Cos7 cells and BHK cells **(I) **were transiently transfected with pMayven-EGFP and actin stained. Mayven is transported to the tips and overlaps in part with actin in all neural cell types **(A-I) **in contrast to the distribution pattern in Cos7 and BHK transfectants **(H,I)**.

Previously we visualised Mayven localization in oligodendroglial lineage cells using an antibody [6] (a gift from S. Avraham), however this was unavailable for further studies. For extended analysis on the subcellular distribution of Mayven, we performed transient transfections on the morphologically plastic and highly process forming OECs. Transfection efficiencies varied between 15 and 40%. Routinely, well over 50% of pMayven-EGFP transfectants had intense regions of EGFP activity positioned towards the process tips overlapping in part with lamellipodial actin (Fig. 3A) similar to the distribution pattern described in OPC transfectants [7]. Variable somal staining was also observed. Soma and process staining was more uniform in transfectants lacking these regions of accumulated Mayven in the tips. This process enriched distribution pattern was also obtained following co-transfection of pMayven-HA and pEGFP-Actin confirming exogenous Mayven transport and accumulation in the process tips is relatively unaffected by the nature or length of the conjugated reporter (Fig. 3A,B). Mayven was clearly absent from the more fine filipodial extensions and does not define the membrane boundary of OEC lamellipodium (Fig. 3A,B). This contrasted to the distribution of exogenous Lasp-1, an actin binding protein in which does demarcate the lamellipodial membrane as depicted in pMayven-HA and pLasp-EGFP co-transfectants (Fig. 3C).

**Figure 4:**
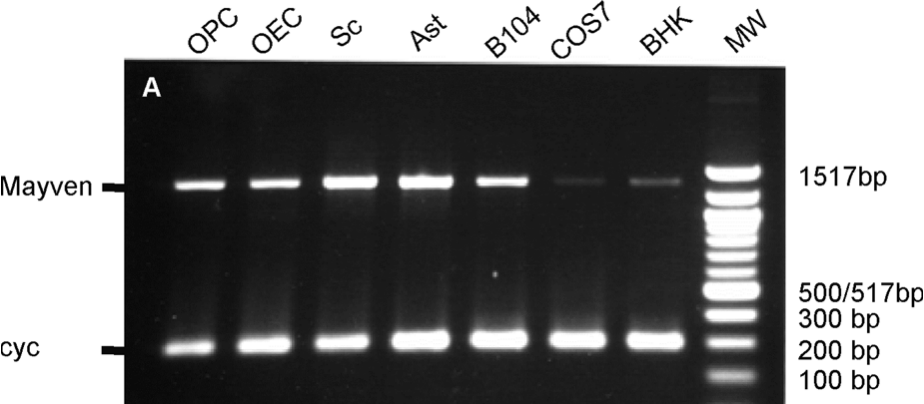
**RT.PCR depicting endogenous *Mayven *message levels in neural and non-neural cells**. *Mayven *(Product length 1323 bps) and *Cyclophilin *(Product length 300 bps) were amplified from cDNAs prepared from O2A c-myc (OPC), (OECs), astrocytes (Ast), Schwann cells (Sc), (B104), (Cos7) and (BHK). *Mayven *mRNA abundance is much higher in neural cell types.

A similar subcellular distribution was also obtained in other neural Mayven transfectants including, Schwann cells, astrocytes, the OPC line Oli-Neu, in addition to the neuroblastoma cell line B104 (Fig. 3D-G). The yellow fluorescence in these merged images depicts a modest and variable degree of overlap between exogenous Mayven and actin towards the process tips. In contrast to neural transfectants, there was no evidence of an accumulation of the fusion protein proximal to cortical actin in transiently transfected Cos7 cells. In this highly vacuolated large rounded fibroblast cell type, staining was more perinuclear and punctate in appearance (Fig. 3H). In transfected BHK fibroblasts which have a more asymmetric cell morphology with processes, Mayven distribution was uniform in nature and did not overlap with cortical actin (Fig. 3I). This stark difference in the distribution pattern between the neural and non-neural transfected cells corresponds to their comparative endogenous *Mayven *mRNA levels. Fig. 4 is a RT.PCR image depicting endogenous message activity relative to the house keeping message *Cycophilin*. *Mayven *mRNA signal intensity in Cos7 and BHK cells (Fig. 4, lanes 6 and 7) is relatively low even at a high cycle number (x35) compared to the message abundance in the neural cells which also display some variation (Fig. 4, lanes 1-5).

**Figure 5:**
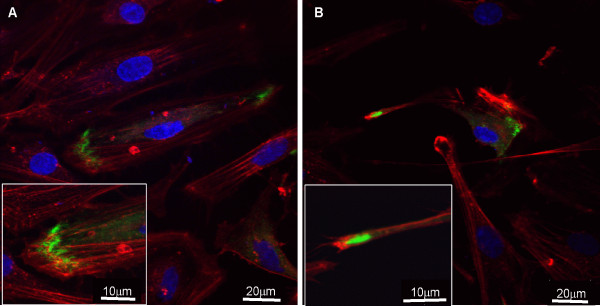
**Cofocal analysis depicting the relationship between the subcellular distribution of Mayven and actin in OEC transfectants**. OECs were transiently transfected with pMayven-EGFP and actin stained. Mayven is transported towards the lamellipodia in both flat **(A) **and **(B) **spindle cells and overlaps in part with actin in both cell types as highlighted in the blow ups.

Confocal micrographs were captured for a more detailed analysis of Mayven and actin localization in OEC transfectants. These essentially mirror the epifluoresence images (Fig. 3) confirming a modest degree of Mayven and actin overlap towards the leading edge of the lamellipodium (Fig 5A,B) suggestive of co-localization of these proteins as reported in OPC transfectants [7]. In addition, the confocal images clearly show that Mayven does not demarcate the lamellipodial membrane.

### Cytoskeletal integrity and the subcellular location of exogenous Mayven

To investigate any relationship between Mayven location in the lammelipodium of OEC transfectants and the integrity of the cytoskeleton, changes in exogenous Mayven distribution was tracked following pharmacological perturbation of the OEC cytoskeleton following treatment with i) Cytochalasin B, ii) Wiskostatin and iii) Nocodazole.

**Figure 6:**
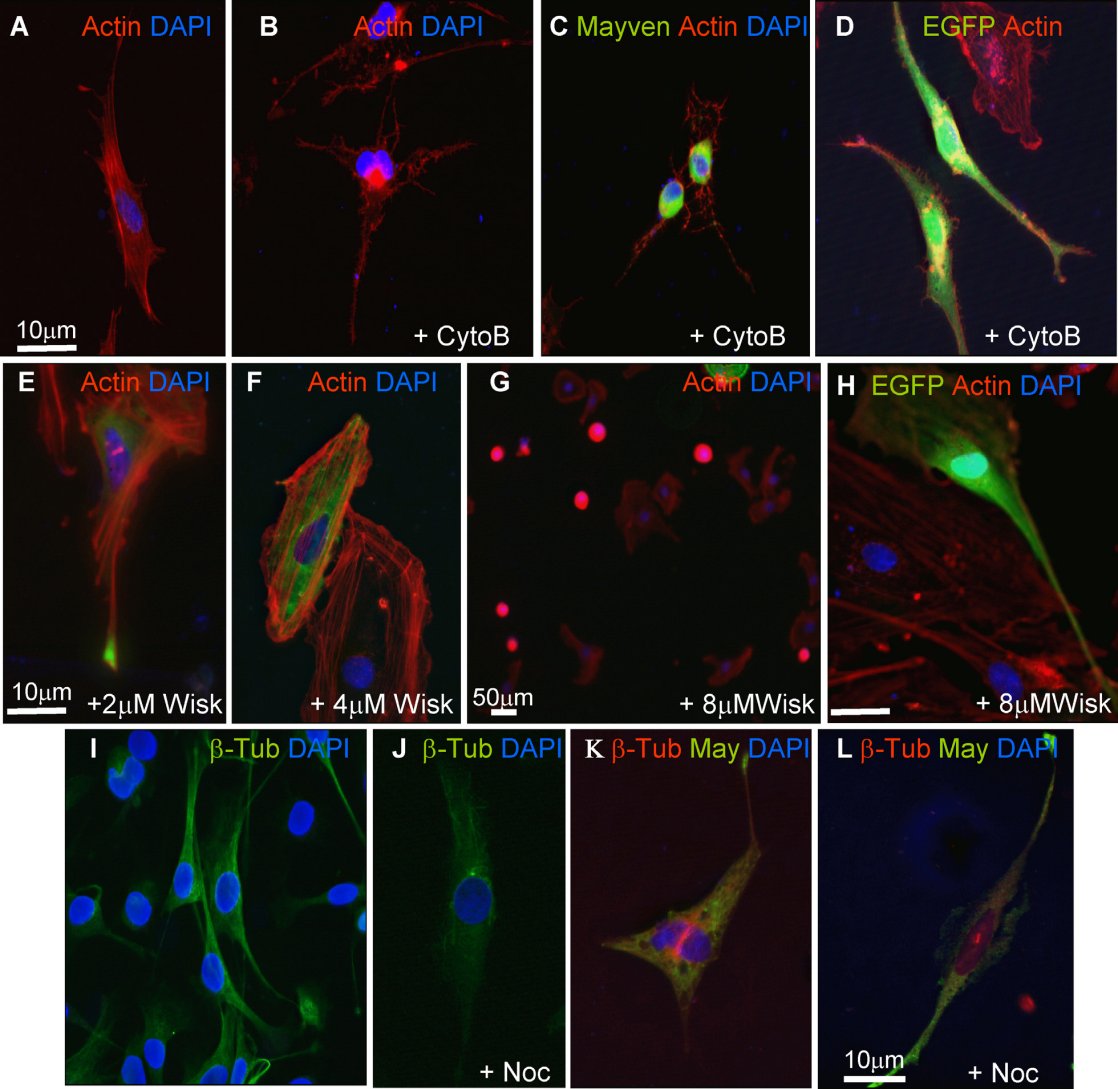
**Subcellular distribution pattern of exogenous Mayven following drug induced disruption of the OEC cytoskeleton with Cytochalasin B (A-D), Wiskostatin (E-H) and Nocodazole (I-L)**. Non-transfected **(A,B) **pMayven-EGFP **(C) **and pEGFP-N1 **(D) **transfected cells were exposed to 0.5 μM Cytochalasin B for 36 hrs **(B-D) **and stained for actin **(A-D). **Cytochalasin B treatment resulted is a redistribution of Mayven **(C) **compared to EGFP **(D)**. Transfected pMayven-EGFP cells were administered 2, 4 and 8 μM Wiskostatin for 2 hrs **(E-G) **and pEGFP-N1 cells were treated for the same duration with 8 μM **(H). **In the absence of cell death there was a relocation of Mayven **(F) **while cell death did not accompany any change in the distribution pattern in pEGFP-N1 transfectants **(H)**. Non-transfected cells **(I,J) **and pMayven-EGFP transfected cells **(K,L) **were treated with 30 μM Nocodazole for up to 3 hrs **(J,L) **and immunostained for β-tubulin **(I-L)**. Mayven loclaization remains unaffected despite a breakdown in the microtubular network **(K,L)**.

i) *Cytochalasin B *Cytochalasin B is a cell permeable fungal toxin that blocks actin monomer addition at the fast growing end of polymers leading to a concentration dependent disruption of the actin cytoskeleton. Song and colleagues reported that treatment of 3 day old OPC cultures with 0.2 μM Cytochalasin B for 36 hrs led to disassembly of the microfilaments accompanied by severely compromised normal process outgrowth and branching [31]. Adopting these guidelines, we found that administration of a 0.5 μM concentration to a one day old OEC culture for 36 hrs resulted in a general breakdown of the actin cytoskeleton, most notably in the processes of the spindle like cells (Fig. 6A,B). Drug treated cells transfected with pMayven-EGFP had a different subcellular distribution pattern from control transfectants with a relocation of Mayven from the process tips to the soma as depicted in the representative image (Fig. 6C). At this Cytochalasin B concentration, the number of process positive staining transfected cells fell to background level of around ≤0.1%. In contrast, there was no observable change in the subcellular location of EGFP in pEGFP-N1 transfected cells, a protein not generally associated with the microfilament component (Fig. 6D). This trend was reproducible (n ≥ 3) suggestive of an intimate relationship between these regions of accumulated Mayven in the process tips and an intact actin cytoskeleton.

*ii) Wiskostatin *Wiskostatin inhibits formation of an active Arp2/3 complex without disrupting the overall integrity of the actin cytoskeleton [32]. Bacon et al [2] had previously shown that administration of 10 μM Wiskostatin to proliferating Schwann cells resulted in the complete retraction of process outgrowth after 30 min. In addition, process extension was fully blocked following drug treatment of freshly seeded Schwann cells. However the specificity of this drug to target Arp2/3 inhibition was the focus of a recent study [33] reporting that at critical concentrations, Wiskostatin treatment also reduces cellular ATP levels leading to an irreversible effect which in turn severely impacts on general cell function including viability.

Taking cognisance of this and using drug concentrations described for Schwann cells [2] pMayven-EGFP and pEGFP-N1 transfected OECs were administered a range Wiskostatin concentrations from 0.5 μM - 10 μM for 2 hrs and assessed for any changes in the Mayven distribution pattern. At concentrations up to 2 μM, there was no evidence of cytotoxicity [33] or any discernible alteration in the integrity of the actin cytoskeleton. At this concentration there was no significant drop in the number of process tip positive cells (Fig. 6E). Although increasing drug level to 4 μM had no measurable cytotoxic effect, it led to a marked reduction in the number of Mayven positive cells compared to 2 μM treated transfectants. In parallel, there was a corresponding increase in the number of somal staining cells indicative of a possible redistribution of the protein from the process tips in drug treated cells (Fig. 6F). At higher drug concentrations (5-10 μM) there was a significant increase in the number of rounded dying cells presumably caused by the non-Arp2/3 effect of Wiskostatin [33] (Fig. 6G). This concentration correlated with the complete absence of process tip positive cells. In contrast to Mayven tranfectants, the distribution pattern in OECs transfected with the control vector pEGFP-N1 was invariant with increasing Wiskostatin concentrations (Fig. 6H). These reproducible findings (n = 3) although not confirming a functional association between Mayven and an active Arp2/3 complex may infer an indirect relationship between inhibition of process extension and Mayven localization.

*iii) Nocodazole *Nocodazole is an anti-mitotic drug that binds to β-tubulin which leads to the inhibition of microtubule assembly. Staining non-treated cells with an acetylated tubulin antibody which binds to an α-tubulin epitope confirmed strong microtubular staining around the perinuclear region and the more proximal areas of the processes (Fig. 6I). Following guidelines we previously described for Nocodzole treatment of Cos7 transfectants [34] we found that OECs exposed to 30 μM Nocodazole for 3 hrs lead to depolymerisation of the microtubular network as evidenced by the breakdown of the characteristic trebecular staining pattern in the vast majority of the treated cells (Fig. 6J). There was no significant change in the subcellular localization of exogenous Mayven in Nocodazole treated transfected population (Fig. 6K,L). This reproducible trend (n ≥ 3) suggests that although the microtubular network provides mechanical stability to the extending lamellipodium, its drug induced disruption of this important cooperative interaction between the microtubular and microfilament component had no discernible effect on the short term localization of Mayven.

### Subcellular distribution pattern following expression of Mayven domain deletion mutants

OECs transfected with pMayven-EGFP gives rise to a well defined subcellular localization pattern characterised by regions of accumulated exogenous Mayven towards the process tips (Fig. 3A; Fig. 5A,B). To identify specific domain elements involved in the transport of exogenous Mayven from the cytoplasm to the process tips, we have tracked the subcellular destination of EGFP tagged Mayven following transient transfection of OECs with a panel of amino and carboxyl domain deletion mutants (Fig. 2(i,ii)). Deletion of the SH3 motif (pΔSH) resulted in a uniform staining pattern (Fig. 2(i),A). Excluding the nucleus, EGFP activity was present throughout the cell body and the proximal end of the processes. The intense regions of staining corresponding to loci of accumulated Mayven along the processes, characteristic of wild type transfectants, were absent. There was no overlap with actin. A similar trend was observed following transfection with pΔFYN and pΔBTB/POZ (Fig. 2(i),B,C). Complete deletion of the amino terminus (pΔBACK) resulted in a nuclear and somal signal pattern accompanied with weak EGFP signal in the proximal end of the processes (Fig. 2(i),D). These findings suggest that the accumulation of exogenous Mayven at the distal end of the processes is dependent upon the presence of the SH3 motif of the Fyn Kinase binding domain. However, expression of the carboxyl mutant pΔKELCH, encoding the complete amino terminus did not produce the wild type pattern, localization of this truncated Mayven protein was nuclear and somal (Fig. 2(ii),E) similar to that observed in pBACK transfectants (Fig. 2(i),D). Expression of recombinants pΔKelch-456 and pΔKelch-6 encoding three and five Kelch units respectively gave a distribution profile (Fig. 2(ii),F,G) resembling the amino mutants pΔSH3, pΔFYN and pΔBTB/POZ (Fig. 2(i), A-C). In conclusion, the accumulation of Mayven in the process tips is dependent on the presence of a SH3 motif and the six units comprising the Kelch domain. This pattern in distribution between wild type and mutant forms was observed in other glial transfectants and in the B104 cell line but not in transfected Cos7 or BHK cells (data not shown).

### Expression of SH3 mutants compromises exogenous Mayven subcellular localization

Expression studies with pΔSH3 demonstrated that complete deletion of the proline rich SH3 motif (PPLPPA) of the putative Fyn Kinase binding domain disrupts the signature wild type distribution pattern (Fig. 2A). To identify critical sequences within the SH3 motif associated with this phenotype, three mutants were assayed (Fig. 2(iii)). Removal of the first three residues (pΔPPL) of the motif resulted in a similar pattern to pΔSH3 (Fig. 2H). A mutant phenotype was also observed following expression of pQA carrying two proline substitutions (**Q**PL**A**PA) in the motif (Fig. 2I). In contrast, transfection with pDK, a mutant with an intact SH3 motif but with a double amino acid substitution between the initiation codon and the SH3 motif gave a localization pattern indistinguishable from wild type Mayven cDNA (Fig. 2J). This trend was maintained in other transfected neural cells but not in Cos7 or BHK transfectants (data not shown).

### Wild type and mutant process length and dynamics

**Figure 7:**
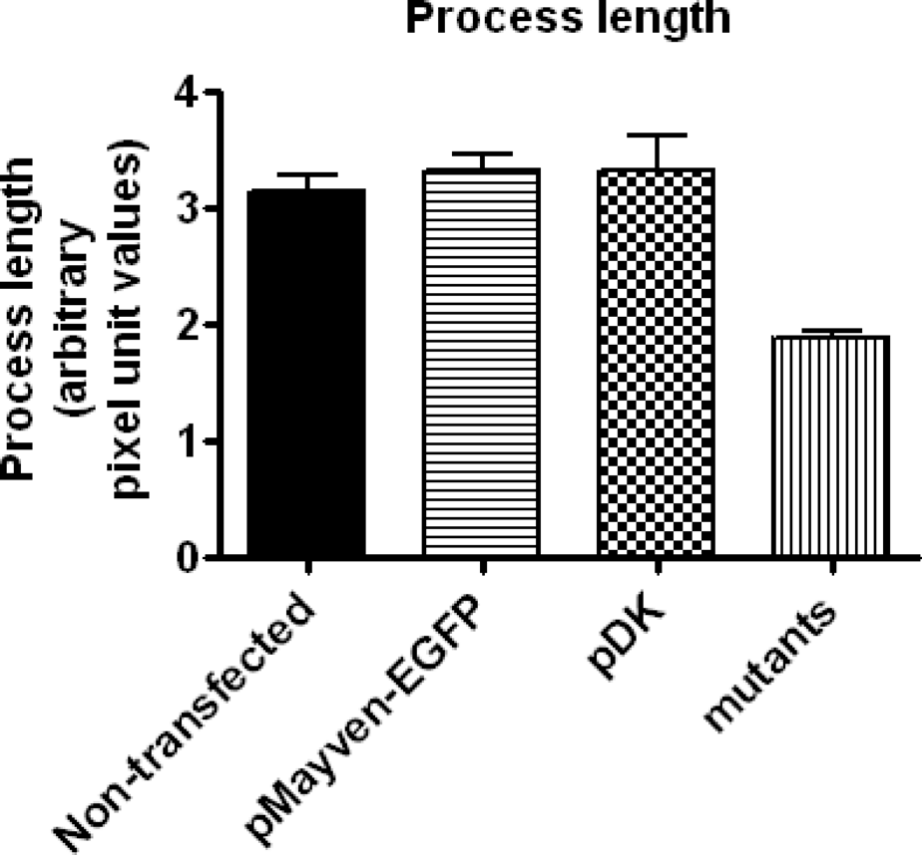
**Relative process length between non-transfected OECs compared to pMayven, pDK, and a pool of mutant transfectants**. Compared to non-transfected cells there is a significant reduction in process length in the pool of mutant transfectants.

A reproducible observation from a series of OEC transfections was that all *Mayven *mutants except pDK had a similar subcellular distribution phenotype (Fig. 2ii) and that these transfectants appeared to have shorter processes than transfectants expressing the wild type form or pDK. For this reason we performed an analysis to quantify this perceived trend. A relative comparison of process length using Image J software was undertaken. The rationale for this pooling strategy was to test the prediction that this common mutant distribution phenotype may reflect a general reduction in process length. Analysis was made from images captured from 75 mutant cells that had successfully been transfected over several experiments and comprised of cells transfected with pQH (7 cells), pΔSH3 (9 cells), pΔFYN (9 cells), pΔBTB/POZ (6 cells), pΔBACK (12 cells), pΔKELCH (11 cells), pΔKELCH-456 (13 cells) and pΔKELCH-6 (8 cells). Transfected and adjacent non-transfected cells with a spindle like morphology with well defined processes were selected. Mutant process length values were pooled and compared to a non-transfected population (23 cells) and pDK (6 cells). As depicted in Fig. 7 there was a statistical significant (P < 0.001) difference of around 40% in process length between non-transfected cells and the pool of mutants. In contrast, a comparison of the non-transfected mean values with those of pMayven-EGFP and pDK did not reveal any significant difference in the average process length between these groupings.

Time lapse recording of Mayven wild type and mutant transfectants are shown in supplemental data. In Additional file 1 OPCs were transfected with pEGFP-N1. EGFP is localized throughout the cell delineating the entire cell and is not concentrated in the processes. Conversely in Additional file 2 a typical OPCs transfected with wild type Mayven shows intense staining in the tips which can be seen to move along the process during time lapse. An example of one of the Mayven mutants (pΔFYN) can be seen in Additional file 3. In this case, Mayven distribution is less intense and is absent from the process tips making the cell appear quite small. These recordings are selected images from the time lapse recording spaced apart by 5 min.

### Differing roles for the BTB/POZ and BACK domains in exogenous Mayven transport

**Figure 8:**
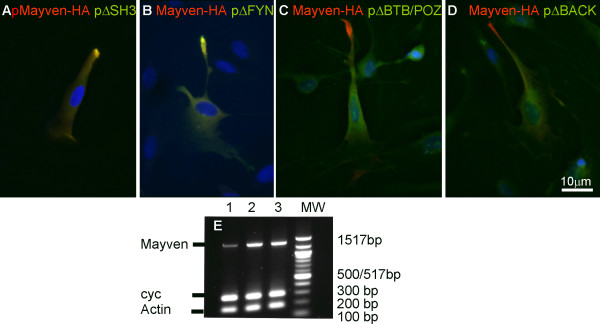
**Subcellular distribution pattern of exogenous Mayven following co-expression of a series of pMayven-EGFP amino deletion mutants with pMayven-HA and RT.PCR comparing endogenous and exogenous *Mayven *message levels in non-transfected and transfected OECs**. OECs were co-transfected with pMayven-HA and mutant constructs pΔSH3 **(A), **pΔFYN **(B), **pΔBTB/POZ **(C) **and pΔBACK and immunostained for HA. Homomeric interaction **(A,B) **between the wild HA tagged fusion protein and EGFP tagged mutant form was dependent on the presence of a BTB/POZ domain. **(E) **RT. PCR *Mayven *(Product length 1323 bps) *Cyclophilin *(Product length 300 bps), β actin (146 bps) were amplified from cDNAs prepared from non-transfected OECs (lane 1), pΔBTB/POZ transfectants (lane 2) and pΔFYN transfectants (lane 3) confirming an increase in *Mayven *mRNA levels in transfected cells.

Co-expression experiments with pMayven-HA cDNA (Texas Red) and the various Mayven -EGFP mutants (FITC) afforded an opportunity to compare the subcellular distribution between wild type and mutant forms of Mayven in the same transfected OEC cell. Transient co-transfection with Mayven-HA cDNA and the SH3 mutant plasmids pΔSH3, pΔPPL, pQA in addition to pΔFYN gave a somewhat unexpected finding in that the EGFP tagged mutant form of the protein overlapped with the HA tagged wild type form as evidenced by the yellow fluorescence in the merged images (Fig. 8A,B) depicting pΔSH3 and pΔFYN co-transfectants respectively. In contrast, there was no evidence of "phenotypic rescue" *via *protein interaction between wild type and mutant forms following co-transfection with pΔBTB/POZ or pΔBACK. Fig. 8C,D shows a strict demarcation of the HA tagged Mayven at the process tips and the EGFP-mutant protein in the cytoplasm. Both of these constructs lack the BTB/POZ domain which has been reported to mediate homo-dimerization of Mayven [8].

However why the proteins encoded by pΔSH3 and pΔFYN mutants are retained in the soma in the presence of endogenous Mayven (Fig. 2(ii)) but are rescued upon supplementation with exogenous HA tagged Mayven as evidenced in the co-transfection experiments (Fig. 8A,B) requires explanation. Although variable, the presence of a high cDNA copy number following a transient transfection generally results in a higher abundance of exogenous protein compared to its endogenous form. This observation in part may explain the apparent failure to affect a "phenotypic rescue" arising from the interaction between endogenous Mayven and the EGFP tagged exogenous proteins encoded by pΔSH3 and pΔFYN plasmids. In co-transfected cells, the abundance of wild type HA-tagged Mayven and EGFP-mutant forms are predicted to around equimolar allowing detectable interaction ie "phenotypic rescue". In contrast, endogenous Mayven levels in mutant transfectants are much lower and consequently are unable to dimerize via their respective BTB/POZ domains in sufficient numbers to elicit a detectable "phenotypic rescue". This is consistent with RT.PCR evidence depicting *Mayven *message levels between control cells, pΔBTB/POZ and pΔFYN transfectants (Fig. 8E lanes 1-3) which show a significant increase in *Mayven *message levels in the transfected cells following gel loading adjustment to the two house keeping mRNAs *Cyclophilin *and β-*Actin*. Moreover, this exogenous:endogenous ratio between transfected and control cells at the individual cell level will be significantly greater than the ratios depicted in the RT.PCR (Fig. 8E) when the transfection rate is considered.

## Discussion

### Exogenous Mayven subcellular localization and the OEC cytoskeleton

Several lines of evidence suggest that Mayven, an actin binding, multi-domain protein member of the BTB-BACK-Kelch family has a key role in oligodendroglial process extension [6,7].

We found that OECs transfected with wild type Mayven-EGFP cDNA gave a similar pattern to the subcellular localization of endogenous Mayven in oligodendrocytes overlapping in part with lammelipodial actin [6,7]. Although OECs are distinct from other macroglia, they share many molecular characteristics with Schwann cells [24,35] and provided appropriate cellular cues are present, OECs can myelinate axons [36]. Furthermore, it has been shown that OECs have morphological plasticity with extensive process formation [3,4]. Allied to the high transfection rate compared to oligodendroglial lineage cells, we consider OECs to be an appropriate cell type to monitor exogenous Mayven transport.

A combination of endogenous and exogenous Mayven and actin co-localization studies, together with a series of co-immunoprecipitations experiments provide compelling evidence that Mayven is an actin binding protein [6-8]. Our Cytochalasin B studies suggest a close association between the integrity of the actin cytoskeletal network and Mayven localization in the process tips. The assembly and depolymerisation of actin filaments and organization into functional higher order networks evident in lamellipodia and filipodia is ultimately regulated by a heterogeneous group of actin binding proteins (ABPs). It is interesting to speculate whether Mayven can be ascribed to a particular class of ABPs from its subcellular distribution pattern. Lamellipodial actin is assembled in part by crosslinking proteins like spectrin and filamin which arrange actin filaments into orthogonal arrays [37]. However, Mayven lacks the typical domain organization associated with this ABP grouping and since Mayven only partially overlaps with lamellipodial actin, may argue against Mayven's inclusion in this class of ABPs. Another group of ABPs for consideration are the sidebinders and signallers such as Cortactin and ENA/VASP [37]. Like Mayven, these are multi-domain proteins that interact with other proteins within signalling networks resulting in cytoskeletal remodelling. The presence of the Fyn Kinase binding domain supports its candidacy for this ABP grouping.

ABPs have a crucial role in actin nucleation *via *formation of the Arp2/3 complex. Immunostaining evidence has confirmed that the Arp2/3 component protein p34 and polypeptides crucial to its activation most notably, N-WASP, WAVEs, Cdc42 and Rac localize close to the oligodendrocyte and Schwann cell membrane [2] contrasting with Mayven's absence from the protruding lamellipodial membrane. We found that treatment of pMayven-EGFP and pEGFP-N1 OEC transfectants with low concentrations of the Arp2/3 inhibitor Wiskostatin, resulted in a redistribution of Mayven compared with an unaltered EGFP pattern. At this Wiskostatin concentration, there was no evidence of cell death arising from the non-specific of the drug leading to a reduction in cellular ATP levels [33].

Although the microfilament and the microtubular networks have distinct functions and a well defined subcellular distribution patterns, there is a growing body of evidence supportive of a coordinated relationship between the two major cytoskeletal components mediated by signalling pathways using common regulators most notably the GTPases [38,39]. However, our findings suggest that depolymerization of the microtubules following administration of Nocodazole had no significant effect on the subcellular distribution of Mayven in OEC transfectants concluding that Mayven is unlikely to be involved in these important cytoskeletal coordinated events.

Following this speculation on Mayven function based on lamellipodial localization, its absence from filipodia and the outer membrane of oligodendrocytes [6,7] may provide inferred evidence to rule out certain biological functions for Mayven. Actin within the filipodia is organized in parallel bundles and provides the initial anchorage points on the axon prior to ensheathment [40] which would seem to uncouple Mayven function from axonal contact.

### Wild type and mutant subcellular distribution pattern

To identify domain or sub-domain regions involved in the subcellular distribution of Mayven we transfected OECs with the panel of EGFP tagged Mayven mutants. Only cells transfected with pDK, which carries two point mutations between the initiation codon and the SH3 motif which gave a wild type distribution pattern. Expression of all other mutants confirmed Mayven was present throughout the soma and in the nucleus of pΔBACK and pΔKELCH transfectants. Signal also extended to the more proximal regions of the processes. This pancellular appearance is similar to the distribution profile observed in the control vector pEGFP-N1 transfectants. This may be regarded as the default transport pattern or non-specific diffusion of this foreign protein. Although fusion proteins derived from expression of mutant cDNAs are present in the processes, they maybe unable to interact with their binding partners as evidenced by the absence of intense staining regions characteristic of the wild type form. This difference in localization may have functional significance. It is intriguing that this relationship between the similarity in the distribution pattern of exogenous Mayven in OECs transfected with wild type and pDK with mutant transfectants mirrors their respective differences in their process lengths. This dominant negative effect observed with our panel of Mayven mutants, is consistent with our earlier study reporting a reduction in process length in OPCs transfected with a c-myc tagged Mayven mutant lacking the SH3 motif [6].

The static transfectant images presented in our study depicting the uniform distribution of exogenous Mayven in OECs transfected with each Mayven mutant (except pDK) contrasts with the characteristic wild type pattern with regions of accumulated Mayven in the process tips. Time-lapse experiments performed with O2A-cmyc wild type transfectants confirm that these regions of accumulated Mayven are intimately involved in process motility and extension. The absence of these accumulated regions in mutant transfectants coincides with compromised motility and extension.

However, our findings are inconsistent with a previous study on the transfection of two amino tagged EGFP mutants in an OPC background [7]. Expression of these truncated constructs, pEGFP-SH3-FYN-BTB/POZ and pEGFP-BTB/POZ lack the BACK-KELCH region which is present in our equivalent recombinants (Fig 6(i),(ii)) co-localized with actin The other main difference between both sets of deletion mutants is the relative orientation of the EGFP moiety. However, we found no difference, following expression of pEGFP-ΔFYN and pΔFYN-EGFP (data not shown). Direct comparison with our findings and this study [7] is difficult as the wild type subcellular distribution pattern is not described.

### Mayven function: Multi-protein complex formation

Although the *Mayven *gene is expressed in a wide range of cell types throughout development, transcript levels are enriched in the CNS with some regional variation in brain tissue [8]. To date, as there is no evidence of splice variants, whether the gene is expressed in the oligodendrocyte, the OEC or the hepatocyte, the Mayven polypeptide is predicted to have the same structural domain organization (Fig. 1A). Thus, it is likely that Mayven's functional diversity is dependent on how the individual domains react and cooperate with binding partners, which will vary with cellular background. This is supported by our observations that exogenous Mayven has a different subcellular localization between neural and non neural cells (Cos7 and BHK fibroblasts). This, allied to low endogenous mRNA levels suggest that Mayven function, if any, in these cells differs from Mayven function in neural cells. Co-localization evidence and immunoprecipitation studies confirm a close association/interaction between Mayven and Fyn Kinase in OPCs [7]. The non-polarized distribution of exogenous Mayven in these fibroblasts maybe related to the relatively low levels of endogenous Fyn Kinase in Cos7 cells [41] and contrasts with the higher abundance in glial cells [42]. Accordingly, this predicted imbalance between exogenous Mayven and endogenous Fyn Kinase in Cos7 transfectants may impact on the subsequent distribution pattern of Mayven and its absence from cortical actin.

A significant increase in *Mayven *gene expression between breast epithelia and corresponding tumour tissue has been reported [43]. This led to an induction of c-jun levels resulting in cell division following cyclin D1 activation *via *the JNK pathway. Using a series of Mayven domain reporter constructs revealed that this cascade was mediated by the BTB/POZ domain. In a more recent study NAC1, which encodes a BTB/POZ domain acts as a co-repressor for other BTB/POZ proteins in the mature CNS was shown to bind to Mayven *via *heteromeric interaction between their respective BTB/POZ domains [44]. Consistent with our findings on the role of the BTB/POZ in Mayven dimerization and in the rescue of Mayven mutant phenotypes, is the study by Soltysik-Espanola and colleagues using a cross-linking agent to assess protein interaction under reducing and non-reducing conditions concluding that Mayven was capable of dimerization in solution [8].

**Figure 9:**
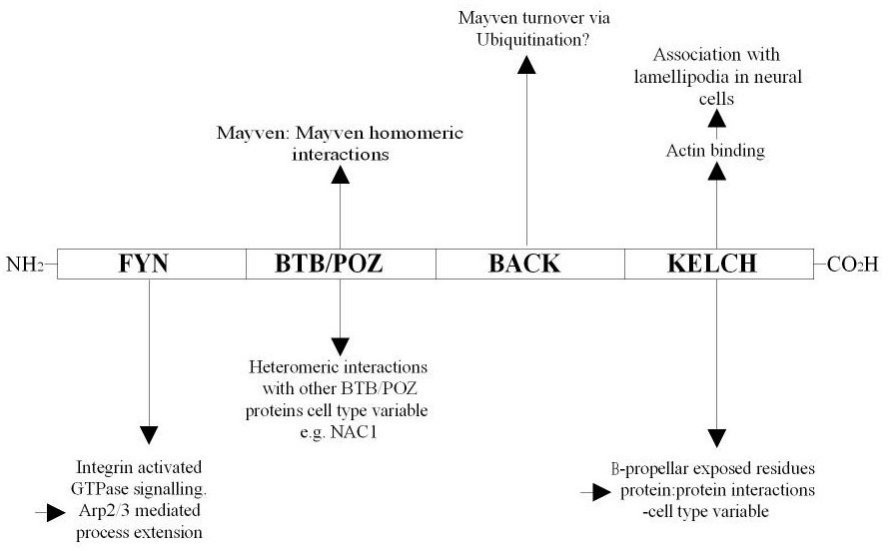
Schematic depicting proposed individual domain function in Mayven multi-protein complex formation

We propose that Mayven localizes to the lamellipodium proximal to a series of other proteins that appear to share a key role in neural process formation by actin polymerization driven membrane protrusion. As depicted in the schematic (Fig. 9) Mayven has a domain organization with the predicted capacity to self-associate *via *its BTB/POZ domain forming Mayven dimers or complex with other BTB/POZ proteins such as NAC1 [44] and interact with higher order actin structures and other binding partners through its Kelch domains to form different multi-protein complexes with a range of as yet unknown function. In a glial background, Mayven may be involved through its Fyn Kinase binding domain, with integrin activated signalling cascades involving Fyn Kinase and the small GTPase family members, RhoA, Rac1 and Cdc42 [19,20] linking extracellular stimuli to changes in the organization of the cytoskeleton *via *Arp2/3 activation.

## Conclusions

Generation of this panel of Mayven mutants has illustrated important properties of three domains in the location of Mayven along cell processes and their possible functional roles in process extension. For example recruitment of Mayven to the leading edge of neural cell lamellipodia requires the presence of a wild type SH3 motif of the putative Fyn Kinase binding domain at the amino terminus and a full complement of the six units comprising the Kelch domain. In addition the BTB/POZ domain appears to be important in homomeric interactions. Lastly the subcellular localization of exogenous Mayven is reliant upon the presence of an intact actin cytoskeleton.

## Authors' contributions

PM generated all the tools for the molecular studies, designed experiments, collected and assembled data and contributed to manuscript writing, SCB carried out the time lapse, designed experiments, contributed to manuscript writing, and approved the manuscript for submission. PGEK contributed to manuscript writing and obtained financial support for the study. All authors read and approved the final manuscript.

## Supplementary Material

Additional file 1 **Time lapse recording of pEGFP O2A-cmyc cells**. Transfectants were visualised over 18 hr period and images collected every three min using a Nikon TE2000 with perfect focus. It can be seen that the entire cell is filled with fluorescence. Click here for file

Additional file 2 **Time lapse recording of pMayven-EGFP O2A-cmyc cells**. Transfectants were visualised over 18 hr period and images collected every three min using a Nikon TE2000 with perfect focus. EGFP Mayven can be seen to move along the tips of the cells. Click here for file

Additional file 3 **Time lapse recording of a SH3 mutant Mayven-EGFP in O2A-cmyc cells**. Transfectants were visualised over 18 hr period and images collected every three min using a Nikon TE2000 with perfect focus. In this mutant Mayven remain perinuclear and can not be seen to be transported in the cell processes. Click here for file
